# P276-00, a cyclin-dependent kinase inhibitor, modulates cell cycle and induces apoptosis *in vitro* and *in vivo* in mantle cell lymphoma cell lines

**DOI:** 10.1186/1476-4598-11-77

**Published:** 2012-10-18

**Authors:** Nitesh P Shirsath, Sonal M Manohar, Kalpana S Joshi

**Affiliations:** 1 Oncology Franchise, Piramal Healthcare Limited, 1-Nirlon Complex, Goregaon, Mumbai, 400 063, India; 2 Target Identification Group, Piramal Healthcare Limited, 1-Nirlon Complex, Goregaon (E), Mumbai, 400 063, India

**Keywords:** Mantle cell lymphoma, Cdk inhibitors, P276-00, Cyclin D1, Mcl-1

## Abstract

**Background:**

Mantle cell lymphoma (MCL) is a well-defined aggressive lymphoid neoplasm characterized by proliferation of mature B-lymphocytes that have a remarkable tendency to disseminate. This tumor is considered as one of the most aggressive lymphoid neoplasms with poor responses to conventional chemotherapy and relatively short survival. Since cyclin D1 and cell cycle control appears as a natural target, small-molecule inhibitors of cyclin-dependent kinases (Cdks) and cyclins may play important role in the therapy of this disorder. We explored P276-00, a novel selective potent Cdk4-D1, Cdk1-B and Cdk9-T1 inhibitor discovered by us against MCL and elucidated its potential mechanism of action.

**Methods:**

The cytotoxic effect of P276-00 in three human MCL cell lines was evaluated *in vitro*. The effect of P276-00 on the regulation of cell cycle, apoptosis and transcription was assessed, which are implied in the pathogenesis of MCL. Flow cytometry, western blot, immunoflourescence and siRNA studies were performed. The *in vivo* efficacy and effect on survival of P276-00 was evaluated in a Jeko-1 xenograft model developed in SCID mice. PK/PD analysis of tumors were performed using LC-MS and western blot analysis.

**Results:**

P276-00 showed a potent cytotoxic effect against MCL cell lines. Mechanistic studies confirmed down regulation of cell cycle regulatory proteins with apoptosis. P276-00 causes time and dose dependent increase in the sub G1 population as early as from 24 h. Reverse transcription PCR studies provide evidence that P276-00 treatment down regulated transcription of antiapoptotic protein Mcl-1 which is a potential pathogenic protein for MCL. Most importantly, *in vivo* studies have revealed significant efficacy as a single agent with increased survival period compared to vehicle treated. Further, preliminary combination studies of P276-00 with doxorubicin and bortezomib showed *in vitro* synergism.

**Conclusion:**

Our studies thus provide evidence and rational that P276-00 alone or in combination is a potential therapeutic molecule to improve patients’ outcome in mantle cell lymphoma.

## Background

Mantle cell lymphoma (MCL), an aggressive B-cell malignancy constitutes about 4-10% of all non-Hodgkin lymphomas (NHLs) population [1]. It exemplifies its clinical onset by a typical gathering of CD20+/CD5+ B cells in lymph nodes, spleen, bone marrow, and blood [2]. Classic (80-90% cases) and blastoid (10-20% cases) are the two powerful variants recognized where the latter is associated with inferior clinical outcome and poor prognosis [1,3-5]. Although treatment with combination chemotherapeutic regimens can be effective, virtually all patients relapse and the outcome of patients remains poor, with a median survival of only 3-5 years [6,7]. Currently available therapies including high-dose chemotherapy followed by stem cell transplant, and monoclonal antibody therapy have shown limited success [2,8]. No therapy has been effective enough to extend the overall survival time of patients with MCL. Thus, it remains incurable with current therapeutics available and awaits more effective treatment approaches [9].

Chromosomal translocation t(11;14)(q13;32) between the IgH and Bcl-1 genes, which results in constitutive overexpression of cyclin D1, represents the hallmark of MCL and seemingly one of the critical oncogenic event, making MCL a genomically highly unstable disease [10-13]. Cyclin D1 coupled with Cdk4 regulates the G1-S transition of the cell cycle and hence this overexpression of cyclin D1 in MCL was thought to contribute to uncontrolled growth. Cyclin D1 overexpression contributes to the lymphomagenesis in MCL by overcoming the suppressor effect that retinoblastoma protein (RB) performs in the G1/S transition [1,14]. RB1 seems to be normally expressed in the majority of MCL cases and the protein appears to be hyperphosphorylated [15], particularly in highly proliferative blastic variants [16]. Enhanced proteolytic degradation of Cdk inhibitors such as p27 and p21 is also observed in MCL [17]. The expression of antiapoptotic members of the Bcl-2 family appears to be one important factor in the acquisition of clinical resistance by MCL cells [18]. From a mechanistic perspective, high levels of expression of the antiapoptotic protein Mcl-1 have been shown to correlate with high-grade morphology and a high proliferative state in MCL [17,19]. In addition, constitutively active STAT3 contributes to the malignant phenotype of MCL by promoting uncontrolled cell growth and survival through dysregulated protein expression, including that of interleukins viz; IL-6 and IL-10 [7].

P276-00, a novel small molecule inhibitor of cyclin-dependent kinases (Cdks), is currently in Phase II clinical trials. It shows better selectivity towards Cdk9-T1, Cdk4-D1 and Cdk1-B as compared with Cdk7-H and Cdk2-E [20,21]. Recently, we showed that it inhibits transcription in multiple myeloma cells by inhibiting Cdk9-T1 which plays a positive regulatory role in transcription [22]. In the present study, we have evaluated and efficacy of P276-00 against MCL. Our hypothesis is that P276-00 being a potent Cdk4-D1 inhibitor will induce rapid cell death in MCL cells which overexpress cyclin D1. Also, its ability to down regulate anti-apoptotic protein Mcl-1 would contribute to its cytotoxic activity for MCL cells. Thus, we provide *in vitro* and *in vivo* evidence for use of P276-00 as a promising therapeutic agent for the treatment of patients with MCL.

## Results and discussion

### Results

#### Cytotoxic potential of P276-00 against MCL

**Figure 1:**
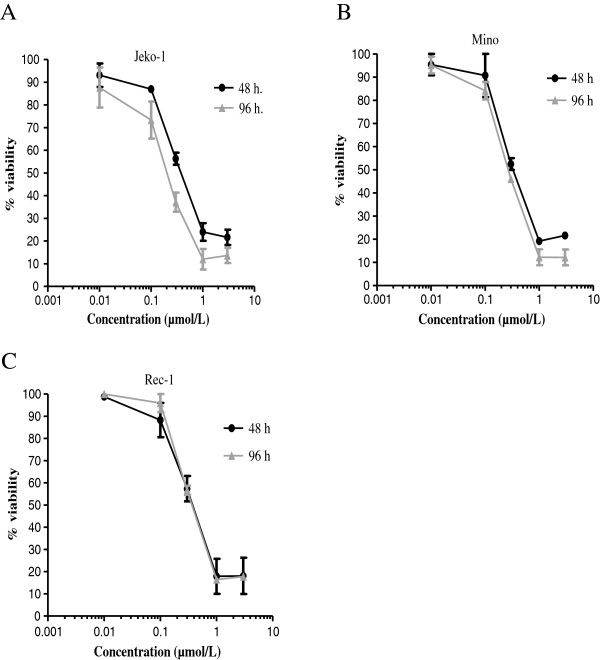
**P276-00 mediated cytotoxic effect in MCL cell lines: Dose and time dependent analysis.** The effect of P276-00 on viability of MCL cells was determined by CCK8 assay. Mantle cell lymphoma cell lines (**A**) Jeko-1, (**B**) Mino, and (**C**) Rec-1 were cultured in the presence of increasing concentrations of P276-00 (0.01-3 μmol/L) for 48 and 96 h. P276-00 showed IC_50_ of 0.35 μmol/L at 48 hr and 0.22 μmol/L at 96 hr in Jeko-1 cells. In Mino and Rec-1 the IC_50_ were 0.5 μmol/L at 48 hr and 0.21-0.33 μmol/L at 96 hr (Table 1). Data presented as the average ± SE of three independent experiments.

All the three MCL cell lines in the presence of increasing concentrations of P276-00 showed significant dose-dependent cytotoxicity as compared to vehicle treated cells (*p* < 0.0001). P276-00 resulted in dose and time dependent cytotoxicity with inhibitory concentration of 50% (IC_50_) ranging from 0.35 μmol/L in Jeko-1 and Mino and 0.5 μmol/L in Rec-1 after 48 h (Figure 1A, B, C, Table 1). Earlier, we have shown that P276-00 is less cytotoxic to resting hPBMCs as compared to conconavalin A (ConA) stimulated hPBMCs [22,23]. These data indicate that P276-00 selectively induces higher cytotoxicity in proliferating cells viz. MCL cells and stimulated hPBMCs, but lesser in quiescent (unstimulated) hPBMCs.

**Table 1:** **IC**_**50 **_**(μmol/L) for P276-00 and roscovitine in Mantle cell lymphoma cell lines**

**Cell lines**	**P276-00**		**Roscovitine**	
	**48 h**	**96 h**	**48 h**	**96 h**
Jeko-1	0.35 ± 0.047	0.21 ± 0.055	25.3 ± 1.83	18 ± 2.82
Mino	0.5 ± 0.28	0.25 ± 0.01	18.1 ± 4.38	14.56 ± 4.55
Rec-1	0.5 ± 0.24	0.33 ± 0.028	32 ± 1.0	40.1 ± 1.27

Jourdan and colleagues [5] reported that interleukin-6 (IL-6) and insulin-like growth factor-1 (IGF-1) aggravate growth and prevent apoptosis in MCL cells. Treatment of MCL cells with P276-00 overcomes this protective effect of IL-6 and IGF-1 as observed with no change in IC_50_s in presence of IL-6 and IGF-1 on MCL cell growth (data not shown).

#### P276-00 inhibited the expression of positive regulators of cell cycle and short-lived oncoproteins

**Figure 2:**
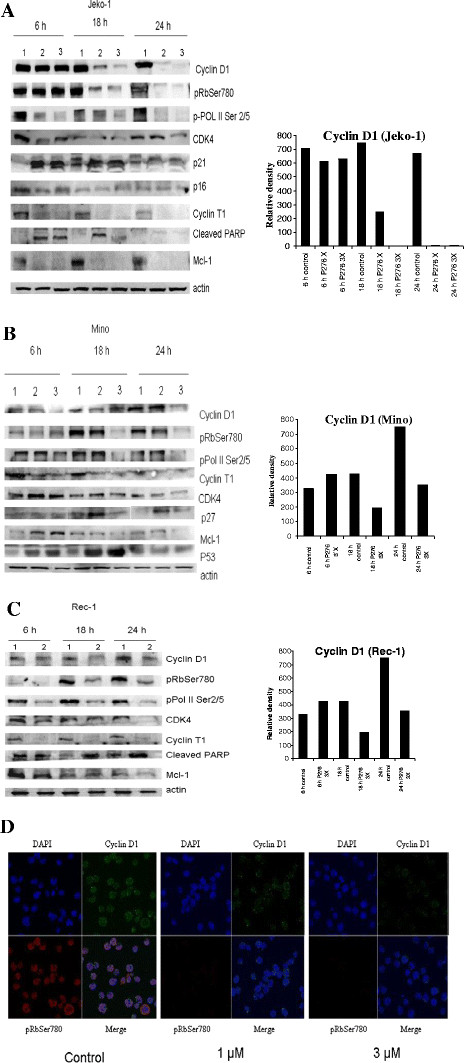
**P276-00 on positive regulators of cell cycle along and anti-apoptotic protein: Western blot analysis of cell cycle proteins at designated time intervals after treatment of (A) Jeko-1, (B) Mino and (C) Rec-1 cells with P276-00.** Jeko-1 and Mino cells (1-control, 2- IC_50_ treated and 3- 3X IC_50_) were incubated with 0.3 and 1 μmol/L P276-00 followed by protein isolation for Western blotting. Rec-1 cells (1-control and 2- treated) were treated at 1.5 μmol/L (3X IC_50_) of P276-00 and it showed marked decrease in all cell cycle related protein level in time dependent manner. Marked down regulation of anti apoptotic protein, Mcl-1, were also seen from early time point in all three cell lines. Densitometric analysis of cyclin D1 expression was done using ImageJ software. (**D**) Regulation of cyclin D1 protein levels and Rb phosphorylation by P276-00 was confirmed by immunofluorescence in Mino after 6 h of treatment. Blue: DAPI (nuclear stain); Green: cyclin D1; Red: pRb^Ser780^.

Previous studies showed that transcriptional inhibition of anti-apoptotic proteins is a key mechanism for Cdk9 inhibitor-induced cell death in indolent B-cell malignancies [22]. P276-00 being a potent inhibitor of Cdk9-T1, we studied its effect on MCL cells. It significantly inhibited the phosphorylation of RNA Pol II CTD 6 h onwards and continued till 18 and 24 h of treatment (Figure 2A, B, and C). Recently, we have shown similar results in multiple myeloma cells and results were attributed to higher selectivity of P276-00 for Cdk9 than Cdk7, which is responsible for serine 5 phosphorylation of CTD of RNA Pol II [20,22]. In MCL cells levels of cyclin T1 were also significantly reduced at all the three time points. Owing to inhibition of transcription, rapid down regulation of a short-lived protein Mcl-1 was observed especially in Jeko-1 cells. P276-00 being a potent Cdk inhibitor we studied its effect on cell cycle proteins and kinases. There was significant down regulation of cyclin D1 protein levels and pRb^Ser780^ which was also confirmed by immunofluorescence in Mino after 6 h of treatment (Figure 2D). Cdk4 levels were decreased in Jeko-1 cells after 6 h and after 24 h in Mino and Rec-1 cells. Interestingly p21 and p27 proteins were found to be increased after P276-00 treatment in Jeko-1 and Mino cells respectively at IC_50_ concentration. Mino cells which harbour wild type p53 showed marked increase in p53 levels (Figure 2B). Increase in the levels of cleaved PARP, a marker for apoptosis was observed in Jeko-1 and Rec-1 cells concomitant with apoptotic cell death. Of all the three cell lines, Rec-1 cell line was found to be the most sensitive. It showed significant down regulation of key proteins such as pRb^Ser780^, cyclin T1 and pRNA pol II^Ser 2/5^ after as early as 6 h of treatment.

#### P276-00 treatment induces apoptosis of MCL cells in a time- and dose-dependent manner

**Figure 3:**
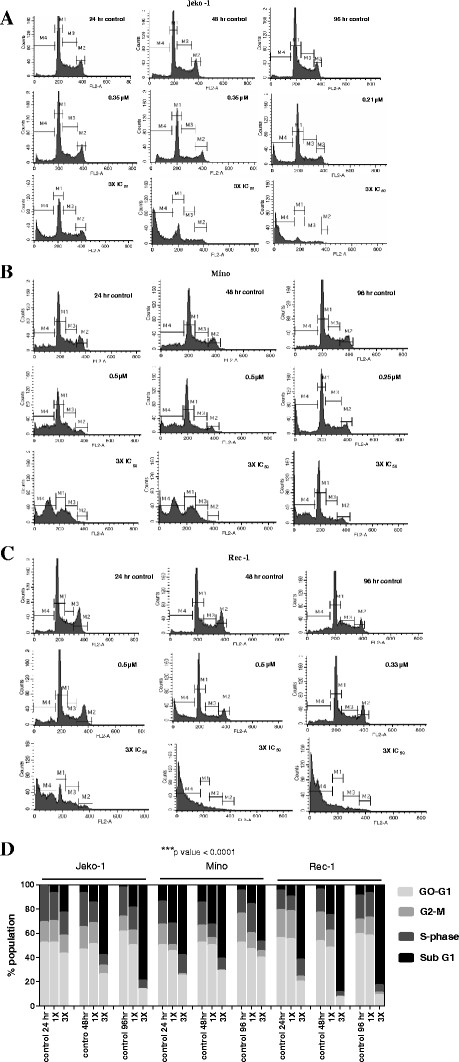
**Effect of P276-00 treatment induces apoptosis in MCL cell lines : Cell-cycle analysis by PI staining was performed on (A) Jeko-1, (B) Mino and (C) Rec-1, cultured with media alone or IC**_**50 **_**and 3X IC**_**50 **_**of P276-00 for the 24, 48, and 96 h time point.** Cells were processed and analyzed by flow cytometry as described in Materials and Methods. Histogram shows shift of cells from G1 phase (M1) to apoptotic phase (M4). Results shown are representative of three independent experiments. Percent change in G1 or S-phase cells was normalized to DMSO vehicle control (**D**) Compiled data shows dose and time dependant increase in apoptotic cells in all three MCL cells (*** *p* < 0.0001)

Cell cycle analysis was performed on MCL variant cells after P276-00 treatment. As demonstrated in Figure 3A, B, C and D P276-00 causes induction of apoptosis in asynchronous population of MCL cell lines when exposed to IC_50_ and 3 times (3X) IC_50_ concentrations. P276-00 resulted in an increase in sub-G1 cells at as early as 24 h with maximal effect noted at 48 and 96 h (70-80% sub-G1 fraction). P276-00 doesn’t allow cells to enter G1 phase and causes significant shift of cells from G_0_-G1 phase to sub-G1 phase.

#### P276-00 rapidly down regulates Mcl-1 transcription

**Figure 4:**
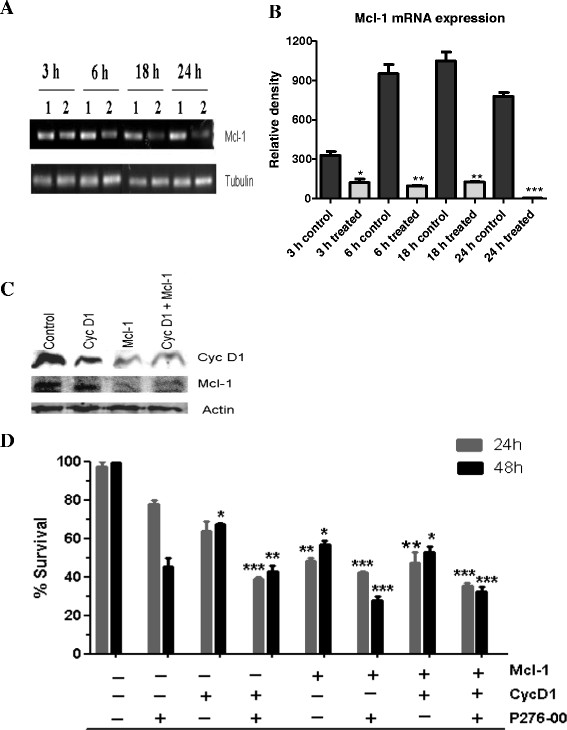
**(A) Mcl-1 mRNA measured by reverse transcription PCR in Jeko-1 cells with (1.5 μmol/L) or without (control) P276-00 treatment at indicated time points (B) Densitometric representation of RT-PCR indicating statistically significant differences compare to control (C) Western blot analysis of protein expression at 48 h after transfection with siRNAs (D) Effect of cyclin D1 and Mcl-1 siRNA in Jeko-1 cells.** Cells were transfected with siRNAs i.e. control siRNA (control) and cyclin D1 (cyc D1) or Mcl-1 or the combination of both the siRNAs (cyclin D1 + Mcl-1) with P276-00. Columns, mean percentage of live cells after transfection (treated) from two independent experiments; bars, SD. **, *** significantly different from control siRNA transfected group (** *p* = < 0.01; *** *p* = < 0.001).

To confirm that the loss of Mcl-1 protein was due to decreased in transcription, the levels of Mcl-1 mRNA following P276-00 treatment were measured by reverse-transcription PCR. Tubulin was used as a control. P276-00 caused a rapid reduction of Mcl-1 mRNA in Jeko-1 cell line from 6 h of treatment with the levels further decreasing up to 24 h. It confirmed that the loss of Mcl-1 protein was due to a block in transcription (Figure 4A, B).

#### Effect of siRNA depletion of Mcl-1 and cyclin D1 on survival of MCL cells

Further we proposed to validate role of cyclin D1 and Mcl-1 in MCL survival using RNA interference (RNAi) approach. siRNAs against Mcl-1 and cyclin D1, which are critical proteins for MCL survival and proliferation, reduced respective protein levels by 60% in Jeko-1 (Figure 4C). There was significant reduction of cell viability at 24 and 48 h with Mcl-1 siRNA and cyclin D1 siRNA at 48 h post transfection. These results highlight the crucial role of Mcl-1 and cyclin D1 in survival of MCL cells. When siRNA treatment was combined with P276-00 significant decrease in percentage survival of cells was observed compared to scrambled siRNA treatment (Figure 4D). Cyclin D1 and Mcl-1 siRNA treated cells in combination with P276-00 showed significant growth reduction at 24 h. At later time point of 48 h, P276-00 with Mcl-1 siRNA showed marked reduction in survival as compared to drug alone (Figure 4D).

#### Anti-tumor effect of P276-00 in xenograft model of MCL

**Figure 5:**
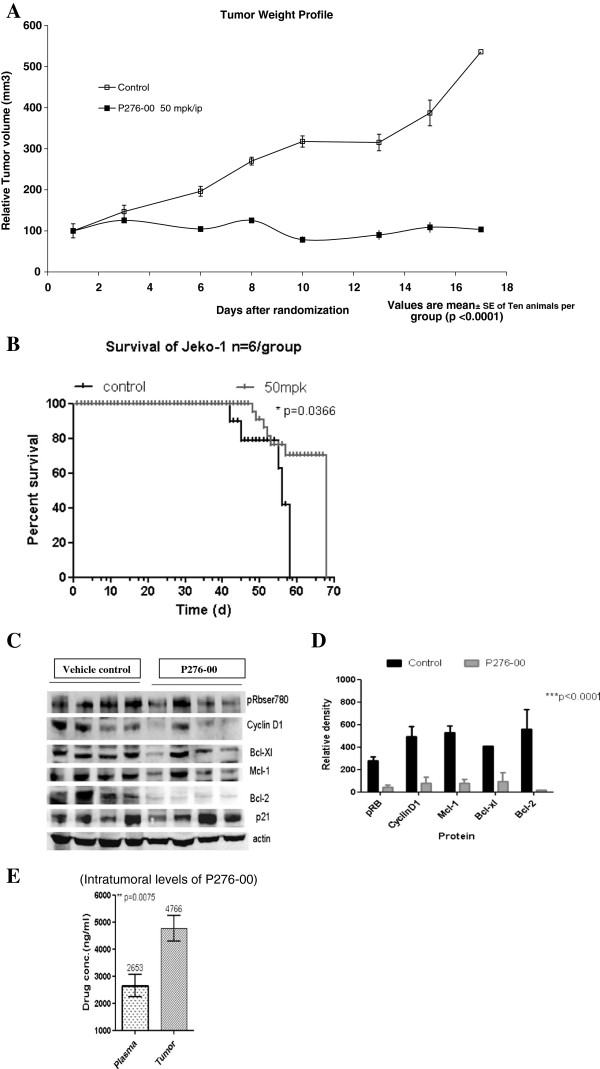
**P276-00 *in vivo *anti-tumor efficacy as single agent and its pharmacokinetics/pharmacodynamic relationship.** (**A**) Treatment with P276-00 in Jeko-1 xenograft by i.p. injection showed significant dose dependent tumor growth inhibition of 91% at 50 mpk with tumor regression on day 8 (***p = 0.0001) and stable disease throughout the schedule (**B**) P276-00 prolonged survival of tumor bearing SCID mice by two weeks compared to untreated shown using Kaplan Meier survival curve (*p = 0.0366) (**C**) Protein expression analysis of the tumor samples showed target engagement with marked inhibition of cell cycle regulating and antiapoptotic proteins (**D**) Densitometric analysis of protein expression in tumor samples showing decrease in levels of proliferation and survival markers (**E**) High intratumoral levels of P276-00-detection by LC-MS for Jeko-1 tumor samples.

*In vivo* P276-00 showed significant tumor growth inhibition of 91% at 50 mg/kg with stable disease throughout the schedule (Figure 5A and B). Kaplan Meier survival curve graph (Figure 5B) showed that mice treated with 50 mg/kg P276-00 (n =10) have a median survival of 68 days (95% confidence interval), which is significantly longer than the median survival of 58 days (95% confidence interval) in control SCID mice. The log-rank test indicated an overall statistically significant difference in survival of P276-00 treated group as compared to vehicle treated group (*p = 0.0366). In PK–PD studies, intratumoral levels of P276-00 reached beyond its effective concentration (Figure 5E) which correlates effectively with significant down regulation of positive regulators of cell cycle (Figure 5C and D).

#### Bortezomib and doxorubicin synergize the cytotoxic effect of P276-00 in MCL

**Figure 6:**
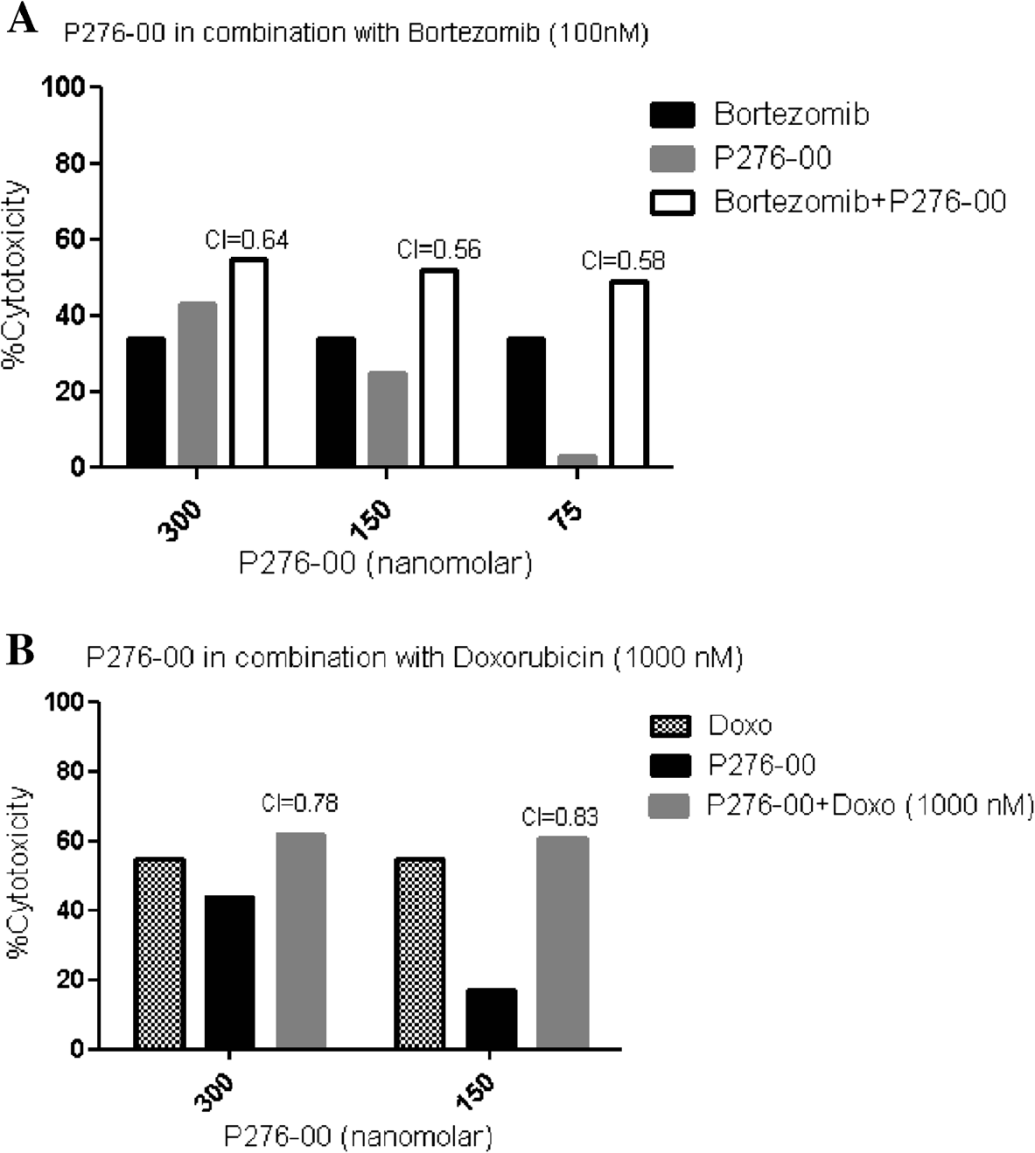
**Combination studies of standard therapies with P276-00.** P276-00 has synergistic anti MCL activity when combined with bortezomib at 100 nM and doxorubicin at 1000 nM. Combination index (CI) was calculated using CompuSyn software. Data for (**A**) Bortezomib (100 nmol/L) and (**B**) Doxorubicin (1 μmol/L) are represented, with synergism noted at given concentration of P276-00 and CI of less than 1.

We next combined P276-00 with bortezomib and doxorubicin at suboptimal doses. Results indicate that the combination was synergistic as studied by Chou-Talalay method to calculate combination index (CI) [24]. Bortezomib (100 nM) and doxorubicin (1000 nM) with P276-00 showed synergism with CI values ranging from 0.56 to 0.83 (Figure 6A and 6B).

## Discussion

Recent advances in the understanding of biology of MCL cells are offering new perspectives for the design of targeted therapeutic strategies. The t(11;14) (q13;q32) translocation occurs in an immature B cell and results in the ectopic and deregulated expression of cyclin D1 and early expansion of tumor B cells in the mantle zone areas of lymphoid follicles. This translocation is considered a primary pathogenesis event that deregulates the cell-cycle control, probably by overcoming the suppressor effect of retinoblastoma 1 (RB1) and cell-cycle inhibitor p27. Since defects in cell cycle regulation and apoptosis are primary events in MCL, small-molecule inhibitors of Cdks may play an important role in the therapy of this disorder. Earlier data from our laboratory has shown that P276-00, a Cdk inhibitor inhibits cyclin D1 and down regulates Cdk4 specific phosphorylation of RB at Ser780 (pRb^Ser780^) along with up regulation of p27 and p21 in breast and lung cancer cell lines [20,21]. Hence, the present study was designed to evaluate the therapeutic implication of P276-00 in MCL.

In this study, we first demonstrated that P276-00 directly inhibited the growth of three MCL cell lines in time and dose dependent manner. It has shown potent cytotoxicity against both nodal and blastic variant MCL cells indicating potential therapeutic implication. Interestingly, our earlier data [20,22] showed that the same treatment did not affect the growth of normal resting hPBMNCs suggesting a good therapeutic window. Further, this compound was able to induce apoptosis and caused an accumulation of cells in G1-S phase of the cell cycle in all three MCL cell lines. This could be due to significant reduction in cell cycle regulators viz. protein levels of cyclin D1, pRb^Ser780^ and Cdk4 along with down regulation of antiapoptotic protein Mcl-1. Earlier studies have also shown that Cdk inhibitors CYC202 and flavopiridol decrease the levels of cyclin D1 in MCL [6]. Moreover P276-00 showed down regulation of an important regulator, pRb^Ser780^ and total Cdk4 which initiates the G1-S transition of the cell cycle. Knockdown of Mcl-1 using siRNA in MCL cells lead to significant apoptosis indicating its importance in cell survival. Similar results have been observed by Chen et al previously [9]. We have shown that in addition to cell cycle protein levels, P276-00 also inhibited transcription of key survival protein Mcl-1 which could be attributed to P276-00 effect on Cdk9 inhibition [22]. Earlier reports indicated that MCL cells use multiple survival pathways to evade apoptosis, which possibly renders them resistant to a variety of therapeutic interventions and hence targeting cyclin D1 alone may not prove to be an effective strategy, especially for MCL blastic variant in which expression of Mcl-1 has been shown to be associated with aggressive phenotype [9,19]. Rapid apoptosis in Jeko-1 cells could be attributed to transcription inhibition of short-lived protein Mcl-1.

In addition to deregulated cell cycle control, it is clear that aberrant apoptotic and pro inflammatory pathways play an important role in pathogenesis of MCL [1]. Therefore, it was interesting to understand combination effects of P276-00 with standard-of-care for MCL. Preliminary combination studies of P276-00 with proteasome inhibitor bortezomib and doxorubicin were found to be synergistic. Further potential combination approaches need to be addressed. Notably, triple combination of siRNA for cyclin D1 and Mcl-1, with P276-00 is significantly effective as compared to drug alone suggestive of the need for inhibition of multiple pathways for proficient therapy for MCL. Of importance, the *in vivo* efficacy in MCL xenograft in SCID mice model demonstrates that P276-00 significantly inhibited tumor growth and prolonged the survival of tumor bearing mice. PK-PD studies on the tumor samples clearly demonstrated down regulation of protein levels for cyclin D1, pRb^Ser780^ along with antiapoptotic proteins viz. Mcl-1 Bcl-2 and Bcl-XL. This indicates that the significant antitumor effect is due to frank apoptosis and it was associated with peak P276-00 plasma and tumor concentration of 5–16 μmol/L in Jeko-1 and Mino tumor samples. Importantly, we observed two times higher parent compound in tumors as compared to plasma indicating that *in vivo* P276-00 is effective and therapeutic to MCL.

## Conclusions

**Figure 7:**
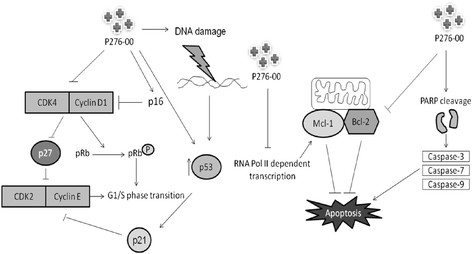
**Schematic representation of effect of P276-00 on cell cycle regulator and apoptosis in MCL.** P276-00, a Cdk4-D1, Cdk1-B and Cdk9-T1 specific inhibitor showed potent antiprolifeartive effect in MCL cell lines by targeting positive and negative regulators of cell cycle with modulation of apoptosis.

In summary, we investigated the action of P276-00, a Cdk inhibitor in three MCL cell lines. Our results show that treatment of MCL cells with P276-00 down regulated important proteins which contribute to pathogenesis of MCL viz. cyclin D1 and Mcl-1 along with cell cycle regulators viz. pRb^Ser780^, Cdk4, Cdk9. These remarkable *in vitro* and *in vivo* efficacies of P276-00, provides a framework for clinical application as a single agent or in combination with conventional therapies in MCL (Figure 7). Thus these data collectively suggest that by merely decreasing the proliferative and survival signatures of the disease we could possibly have a better overall prognosis of the disease. A phase II study is currently ongoing (http://www.seattlecca.org/clinical-trials/lymphoma-UW09052.cfm).

## Methods

### Cell culture and reagents

Human MCL cell lines Jeko-1, Mino and Rec-1 were obtained from ATCC (Rockville, MD, USA). All three cell lines were cultured in RPMI-1640 medium containing 10% fetal bovine serum (FBS) (Hyclone, UT, USA), 2 mmol/L L-glutamine (Gibco, Grand Island, NY, USA), 100 U/mL penicillin and 100 mg/mL streptomycin (Gibco). Cells were maintained at 37°C in a humidified atmosphere containing 5% CO_2_. P276-00 was synthesized at Piramal Healthcare Limited, Mumbai, India, Roscovitine was purchased from Sigma (St Louis, MO, USA). Both drugs were dissolved in dimethyl sulfoxide (DMSO) at a concentration of 10 mmol/L and stored at -20°C until use; required dilutions were made in culture medium RPMI-1640 immediately before use. All reagents were purchased from Sigma (St. Louis, MO, USA) unless stated otherwise.

### *In vitro* cytotoxicity assay

Cytotoxicity of P276-00 on MCL cell lines was assessed using a CCK-8 assay according to the manufacturer’s instructions (Dojindo), as mention earlier [22]. Each concentration was plated in triplicate. P276-00 was applied at five concentrations (0.01, 0.1, 0.3, 1, and 3 μmol/L) while roscovitine was at concentrations (1, 3, 10, 30 and 50 μmol/L). Cells were incubated for 48 and 96 h. At the end of incubation period, CCK-8 was added (10 μL per well) and absorbance was measured at 450 nm using a spectramax microplate reader (Molecular devices, CA, USA). Data was analyzed to determine the IC_50_ (concentration of compound that inhibited cell growth by 50%).

### Preparation and analysis of cell lysates by immunoblotting

The human MCL cell lines were seeded at 1.5 × 10^6^ cells/mL in T-25 flasks and treated with or without IC_50_ or 3X IC_50_ of P276-00 for various time points. Lysates were prepared and western blotting was carried out as described previously [22]. Following antibodies were used: RNA polymerase II CTD phosphoserine 2/5 and Mcl-1 (Cell signaling technology, USA), PARP and Bcl-XL (BD pharmingen, USA), β-actin (Sigma, MO, USA), Cdk4, pRb^Ser780^, p21, p16, p27, p53, Bcl-2, cyclin D1, cyclin T1, anti-rabbit–HRP and anti-mouse-HRP secondary antibodies (Santa Cruz Biotechnology, CA, USA).

### Immunofluorescence and confocal laser microscopy

Mino cells were seeded at a density of 1×10^6^ cells per well of six well plate with treated cells exposed to 1.5 μmol/L P276-00 for 6 h. Cells were harvested and processed for immunofluorescence as described previously [22].

### Analysis of cell cycle distribution by flow cytometry

The human MCL cell lines were seeded in T-25 tissue culture flasks at a density of 0.5 × 10^6^/mL and treated with or without (control) IC_50_ and 3X IC_50_ concentrations of P276-00 for 24 h, 48 h and 96 h. Cells were harvested and processed for flow cytometry as described previously [21].

### RNA extraction and reverse transcription-PCR

Cells were treated and harvested identically to those prepared for immunoblotting. Cell pellets were lysed and RNA was extracted using RNAeasy kit (Qiagen, Valencia, USA). Purified RNA was quantitated and assessed for purity by UV spectrophotometry. cDNA was synthesized from 2 μg RNA with Superscript III reverse transcriptase (Qiagen). The amplification of each specific RNA was performed in a 20 μL reaction mixture containing 2 μL of cDNA template, PCR master mix and the primers. The sequences of the primers were as follows:

Mcl-1 Forward: TAAGGACAAAACGGGACTGG; Reverse: ACATTCCTGATGCCACCTTC with annealing temperature of 55°C and cycle no. 32, Tubulin Forward: TCTGTTCGCTCAGGTCCTTTTGGCC; Reverse: CGTACCACATCCAGGACAGA with annealing temperature of 55°C and cycle no. 32. The PCR products were loaded onto 2% agarose gels and visualized with ethidium bromide under UV light. As a control for cDNA synthesis, reverse transcription-PCR was also performed using primers specific for tubulin gene.

### siRNA mediated RNA interference

Jeko-1 cells were plated in six-well plates with 0.2 × 10^6^ per well in FBS-free and antibiotic-free media. The cells were transfected with siRNA (cyclin D1-specific siRNA or Mcl-1 siRNA or non-specific siRNA, QIAGEN, USA) using Lipofectamine2000 Transfection Reagent (Invitrogen, Carlsbad, CA) as per manufacturer’s instructions. For cyclin D1 and/or Mcl-1 knockdown, the cells were treated with 100 nmol/L siRNA. After transfection, the next day P276-00 was added (1 μmol/L) and the cells were incubated further for another 24 h or 48 h.

### Tumor xenograft model

Severe combined immunodeficient (SCID) mice were injected in 0.2 mL volume (0.1 mL of the cell suspension containing 1 × 10^7^ cells and 0.1 mL of Matrigel) s.c. on the right flank. When the tumors attained a diameter of 100 mm^3^, mice were randomized into 2 groups i.e vehicle control (water) and P276-00 (50 mg/kg). Both the groups were dosed i.p. with vehicle control and P276-00, formulated in water, daily for 15 consecutive days. Animal survival was plotted using Kaplan Meier survival curve and was monitored for 5 weeks post treatment discontinuation. Tumors from animals were excised at the end of the treatment post 15 min of dosing. Further tumors were weighed and snap-frozen for protein expression analysis and pharmacokinetic studies. Protein extracts were prepared and subjected to western blot analysis as explained earlier, and probed with different antibodies. Densitometric analysis of western blots was carried out using Image J 1.42 q software. Animals were maintained and experiments were carried out as per the institutional animal ethical committee in compliance with the guidelines of the Committee for the Purpose of Control and Supervision on Experiments on Animals (CPCSEA), India.

### Statistical analysis

Statistical comparison was made using GraphPad PRISM® (versions 3.0 and 5.0, GraphPad Software, Inc., USA) software where one-way and two way analysis of variance (ANOVA) and Tukey’s multiple comparison post-tests were used to determine significant differences between several treatment groups. Student’s unpaired *t*-test was employed when only two groups were compared. Data are presented as mean ± S.E.M. of at least three independent experiments with triplicates. Statistical significance was evaluated by calculating *p* values, where *p* < 0.05 was considered statistically significant. (*p < 0.05; **p < 0.01, ***p < 0.001). Log-rank test was used for the animal survival study and Kaplan-Meier survival curves were generated from this test.

## Abbreviations

PKPD: Pharmacokinetic pharmacodynamic; Cdk: Cyclin-dependent kinase; RB: Retinoblastoma; hPBMNCs: Human peripheral blood mononuclear cells; mg/kg: Milligram per kilogram; LC MS: Liquid chromatography mass spectrometers.

## Competing interests

The authors declare that they have no competing interests.

## Authors’ contributions

KJ conceptualized and guided the research project. NS performed experiments viz. cytotoxicity, flow cytometry, *in vivo* efficacy including PKPD studies and combination studies. SM performed and analyzed western blot, RT-PCR and siRNA experiments. Manuscript was written by NS and KJ. All authors approved the final manuscript.

## References

[B1] Jares P Campo E Advances in the understanding of mantle cell lymphoma Br J Haematol 2008 142 149 165 10.1111/j.1365-2141.2008.07124.x 18410453

[B2] Liu Q Alinari L Chen CS Yan F Dalton JT Lapalombella R Zhang X Mani R Lin T Byrd JC Baiocchi RA Muthusamy N FTY720 shows promising in vitro and in vivo preclinical activity by down modulating cyclin D1 and phospho-Akt in mantle cell lymphoma Clin Cancer Res 2010 16 3182 3192 10.1158/1078-0432.CCR-09-2484 20460491 PMC4180653

[B3] Ghielmini M Zucca E How I treat mantle cell lymphoma Blood 2009 114 1469 1476 10.1182/blood-2009-02-179739 19556426

[B4] Norton AJ Matthews J Pappa V Shamash J Love S Rohatiner AZ Lister TA Mantle cell lymphoma: natural history defined in a serially biopsied population over a 20-year period Ann Oncol 1995 6 249 256 7612490 10.1093/oxfordjournals.annonc.a059154

[B5] Jourdan M De Vos J Mechti N Klein B Regulation of Bcl-2-family proteins in myeloma cells by three myeloma survival factors: interleukin-6, interferon-a and insulin-like growth factor 1 Cell Death Differ 2000 7 1244 1252 10.1038/sj.cdd.4400758 11175262 PMC2423422

[B6] Venkataraman G Maududi T Ozpuyan F Bahar HI Izban KF Qin JZ Alkan S Induction of apoptosis and down regulation of cell cycle protein in mantle cell lymphoma by flavopiridol treatment Leuk Res 2006 30 1377 1384 10.1016/j.leukres.2006.03.004 16624404

[B7] Baran-Marszak F Boukhiar M Harel S Laquillier C Roger C Gressin R Martin A Fagard R Varin-Blank N Ajchenbaum-Cymbalista F Ledoux D Constitutive and B-cell receptor-induced activation of STAT3 are important signaling pathways targeted by bortezomib in leukemic mantle cell lymphoma Haematologica 2010 95 1865 1872 10.3324/haematol.2009.019745 20663948 PMC2966908

[B8] Goy A Feldman T Expanding therapeutic options in mantle cell lymphoma Clin Lymphoma Myeloma 2007 7 S184 S191 17877843 10.3816/clm.2007.s.021

[B9] Chen R Chubb S Cheng T Hawtin RE Gandhi V Plunkett W Responses in mantle cell lymphoma cells to SNS-032 depend on the biological context of each cell line Cancer Res 2010 70 6587 6597 10.1158/0008-5472.CAN-09-3578 20663900 PMC2929954

[B10] Pileri SA Falini B Mantle cell lymphoma Haematologica 2009 94 1488 1492 10.3324/haematol.2009.013359 19880776 PMC2770958

[B11] Camacho E Hernandez L Hernandez S Tort F Bellosillo B Bea S Bosh F Montserrat E Cardesa A Fernández PL Campo E ATM gene inactivation in mantle cell lymphoma mainly occurs by truncating mutations and missense mutations involving the phosphatidylinositol-3 kinase domain and is associated with increasing numbers of chromosomal imbalances Blood 2002 99 238 244 10.1182/blood.V99.1.238 11756177

[B12] Hangaishi A Ogawa S Qiao Y Wang L Hosoya N Yuji K Imai Y Takeuchi K Miyawaki S Hirai H Mutations of Chk2 in primary hematopoietic neoplasms Blood 2002 99 3075 3077 10.1182/blood.V99.8.3075 11949635

[B13] Tort F Hernandez S Bea S Martinez A Esteller M Herman JG Puig X Camacho E Sanchez M Nayach I Lopez-Guillermo A Fernandez PL Colomer D Hernandez L Campo E CHK2-decreased protein expression and infrequent genetic alterations mainly occur in aggressive types of non-Hodgkin lymphomas Blood 2002 100 4602 4608 10.1182/blood-2002-04-1078 12393693

[B14] de Boer CI Krieken J Schuuring E Kluin PM Bcl-1/cyclin D1 in malignant lymphoma Ann Oncol 1997 8 109 117 9209653

[B15] Zukerberg LR Benedict WF Arnold A Dyson N Harlow E Harris NL Expression of the retinoblastoma protein in low-grade B-cell lymphoma: relationship to cyclin D1 Blood 1996 88 268 276 8704183

[B16] Jares P Campo E Pinyol M Bosch F Miquel R Fernandez PL Sanchez-Beato M Soler F Perez-Losada A Nayach I Mallofre C Piris MA Montserrat E Cardesa A Expression of retinoblastoma gene product (pRb) in mantle cell lymphomas. Correlation with cyclin D1 (PRAD1/CCND1) mRNA levels and proliferative activity Am J Pathol 1996 148 1591 1600 8623927 PMC1861570

[B17] Paoluzzi L Scotto L Marchi E Zain J Seshan VE O’Connor OA Romidepsin and belinostat synergize the antineoplastic effect of bortezomib in mantle cell lymphoma Clin Cancer Res 2010 16 554 565 10.1158/1078-0432.CCR-09-1937 20068080

[B18] Bertoni F Ponzoni M The cellular origin of mantle cell lymphoma Int J Biochem Cell Biol 2007 39 1747 1753 10.1016/j.biocel.2007.04.026 17574898

[B19] Khoury JD Medeiros LJ Rassidakis GZ McDonnell TJ Abruzzo LV Lai R Expression of Mcl-1 in mantle cell lymphoma is associated with high-grade morphology, a high proliferative state, and p53 overexpression J Pathol 2003 199 90 97 10.1002/path.1254 12474231

[B20] Joshi KS Rathos MJ Joshi RD Sivakumar M Mascarenhas M Kamble S Lal B Sharma S In vitro antitumor properties of a novel cyclin-dependent kinase inhibitor, P276-00 Mol Cancer Ther 2007 6 918 925 10.1158/1535-7163.MCT-06-0613 17363486

[B21] Joshi KS Rathos MJ Mahajan P Wagh V Shenoy S Bhatia D Chile S Sivakumar S Maier A Fiebig HH Sharma S P276-00 a novel cyclin-dependent inhibitor induces G1–G2 arrest, shows antitumor activity on cisplatin-resistant cells and significant in vivo efficacy in tumor models Mol Cancer Ther 2007 6 926 934 10.1158/1535-7163.MCT-06-0614 17363487

[B22] Manohar SM Rathos MJ Sonawane V Rao SV Joshi KS Cyclin-dependent kinase inhibitor, P276-00 induces apoptosis in multiple myeloma cells by inhibition of Cdk9-T1 and RNA polymerase II-dependent transcription Leuk Res 2011 35 821 830 10.1016/j.leukres.2010.12.010 21216463

[B23] Manohar SM Padgoankar A Jalota-Badhwar A Rao SV Joshi KJ Cyclin-dependent kinase inhibitor, P276-00, inhibits HIF-1α and induces G2/M arrest under hypoxia in prostate cancer cells Prostate Cancer Prostatic Dis 2012 15 15 27 10.1038/pcan.2011.51 22083267

[B24] Chou TC Drug combination studies and their synergy quantification using the chou-talalay method Cancer Res 2010 70 440 446 10.1158/0008-5472.CAN-09-1947 20068163

